# For U.S. Black women, shift of hysterectomy to outpatient settings may have lagged behind White women: a claims-based analysis, 2011–2013

**DOI:** 10.1186/s12913-017-2471-1

**Published:** 2017-08-04

**Authors:** Whitney R. Robinson, Mariah M. Cheng, Annie Green Howard, William R. Carpenter, Wendy R. Brewster, Kemi M. Doll

**Affiliations:** 1 Lineberger Comprehensive Cancer Center, University of North Carolina at Chapel Hill, Chapel Hill, USA; 2 Department of Epidemiology, UNC Gillings School of Global Public Health, University of North Carolina at Chapel Hill, CB #7435, Chapel Hill, NC 27599-7435 USA; 3 Carolina Population Center, University of North Carolina at Chapel Hill, Chapel Hill, USA; 4 Department of Biostatistics, UNC Gillings School of Global Public Health, University of North Carolina at Chapel Hill, Chapel Hill, USA; 5 Department of Health Policy and Management, UNC Gillings School of Global Public Health, University of North Carolina at Chapel Hill, Chapel Hill, USA; 6 Center for Women’s Health Research, University of North Carolina at Chapel Hill, Chapel Hill, USA; 7 Department of Obstetrics and Gynecology, University of Washington, Seattle, USA

**Keywords:** De-implementation, Health disparities African-American, Gynecology, Administrative data, Ambulatory surgery, Claims-based data

## Abstract

**Background:**

Hysterectomy is among the most common surgeries performed on U.S. women. For benign conditions, minimally invasive hysterectomy is recommended, whenever permitted by clinical indication and previous surgery history. No study has examined whether the use of less invasive hysterectomy spread more slowly for Black women.

**Methods:**

We used the hysterectomy that occurs in outpatient settings as a proxy for minimally invasive hysterectomy. Using claims-based surgery data and census denominators, we calculated age-standardized rates of all hysterectomies in North Carolina from 2011 to 2013. Study participants were 41,899 women (64.6% non-Hispanic White, 28.3% non-Hispanic Black) who underwent hysterectomy for non-malignant indications. We fit Poisson models to determine whether changes in outpatient hysterectomy rates differed by Black-White race. We employed a difference-in-difference approach to control for racial differences in the severity of clinical indication. Further, we restricted to one state to minimize confounding from geographic differences in where Black and White women live.

**Results:**

From 2011 to 2013, the overall hysterectomy rate decreased from 42.3 per 10,000 women (*n* = 14,648) to 37.9 per 10,000 (*n* = 13,241) (*p* < 0.0001). Most hysterectomy (67.6%) occurred in outpatient settings. The inpatient rate decreased 35.2% (*p* < 0.0001), to 10.3 per 10,000, while the outpatient rate increased 4.6% (*p* < 0.01), to 27.5 per 10,000. From 2011 to 2013, Black women’s outpatient rate increased 22% (*p* < 0.0001): from 25.8 per 10,000 to 31.5. In contrast, among White women, outpatient rates remained stable (*p* = 0.79): at 28.3 per 10,000 in 2013.

**Conclusions:**

Rapid increases in outpatient hysterectomy among Black women compared to stable rates among White women indicate a race-specific catch-up phenomenon in the spread of minimally invasive hysterectomy. These results are consistent with the hypothesis that minimally invasive hysterectomy may have been adopted more slowly for Black women than their White counterparts after its introduction in the early 2000s. The persistently high rates of hysterectomy among young Black women and potentially slower adoption of minimally invasive procedures among these women highlight a potential racial disparity in women’s healthcare.

## Background

Hysterectomy is the second most common surgery performed on U.S. women aged 18–64 years, second only to cesarean section [1]. The American College of Obstetrics and Gynecology recommends that benign hysterectomy be done in a minimally invasive fashion, whenever permitted by clinical indication and previous surgery history, because of the known benefits of shorter recovery and equivalent outcomes [2]. A major gap in the hysterectomy literature concerns racial differences in use of minimally invasive hysterectomy. Like other medical innovations, minimally invasive surgery may be adopted unequally by patient social status. If so, older surgical techniques may be “de-implemented” more quickly among socially advantaged people in favor of minimally invasive surgery; then the older techniques would become increasingly concentrated among the racial minorities and other socially disadvantaged groups [3].

Investigating racial differences in the spread of less invasive hysterectomy is important because Black women are disproportionately likely to be treated with hysterectomy [4, 5]. Analysis of nationally representative 2012 CDC data shows marked racial variation in prevalence of hysterectomy: among women aged 48–50 years old, 33% of Black women, 23% of White women, 22% of Hispanic women, and 9% of Asian-American women reported past hysterectomy [6]. Even after adjustment for Black women’s higher rates of leiomyoma diagnoses, Black-White differences in hysterectomy use remained [7, 8]. Further, the state-sponsored eugenics programs of the 20th century targeted hysterectomy disproportionately towards poor women and women of color [9]. Even today, Black women and low-SES women with benign diagnoses are more likely to be treated with hysterectomy than non-Black and higher SES women [10, 11].

Since the early 2000s, hysterectomy has largely shifted to outpatient settings among commercially insured women in the United States [12]. Hysterectomy that occurs in outpatient versus inpatient settings can serve as a proxy for minimally invasive hysterectomy. Unfortunately, the data on commercially insured women are not able to examine racial differences or women insured by Medicaid. A recent analysis of state-based administrative data found marked racial differences in the percentage of procedures occurring in outpatient versus inpatient settings [5]. However, this pooled cross-sectional analysis of 13 states could not address whether these racial differences were mostly likely due to health care system factors, racial differences in clinical indication or geographic differences in where Black and White women live (hysterectomy rates vary widely by U.S. region).

The objective of this paper was to evaluate racial differences in the spread of less invasive hysterectomy to clinically appropriate women. Because information on who is clinically appropriate (based on clinical severity, comorbidities, and other factors) is not available without expensive clinical review of patients or medical records, we employed a modification of the difference-in-difference analytical approach [13]. We compared changes in rates of outpatient hysterectomy over a three-year period between Black and White women. This approach affords three benefits. First, secular trends among White women serve as a control for state healthcare conditions that could influence hysterectomy trends among Black women. Second, we control for confounding by racial differences in clinical indication by examining secular changes in surgery type over a short time period; because clinical indication is not expected to change substantially over three years, we minimized the influence of clinical indication. Third, we focus on outpatient surgery instead of inpatient surgery because declines in inpatient surgery among minority women could be constrained by greater clinical severity and complexity among these women; on the other hand, an analysis comparing increases in outpatient surgery rates for Black versus White women would not be biased by greater clinical severity and complexity among Black women.

## Methods

### Data

Surgery data were obtained from 2 sets of administrative databases collected by the state of North Carolina: the North Carolina Hospital Discharge Data and the North Carolina Ambulatory Surgery Visit Data (Truven Health Analytics, Fiscal Years 2011–2013). For the present analysis of 2011–2013 data, six databases were used: three Discharge databases for fiscal years 2011, 2012, and 2013, and three Ambulatory databases, for the same years. Each fiscal year, data from October 1 of the previous calendar year to September 30 of that calendar year, i.e., fiscal year 2011 extends from October 1, 2010, until September 30, 2011. Unless otherwise, specified “years” refer to fiscal years. Derived from billing data, the Discharge Databases enumerate each inpatient surgery performed in North Carolina in a given year. Similarly, the Ambulatory Surgery Visit database records all outpatient surgery conducted in the state. We define an outpatient surgery as one that was conducted in a free-standing ambulatory surgery center or in which the patient was discharged in less than 24 h, regardless of surgery location. Beginning in 2011, the Ambulatory files include all reported hospital outpatient procedures (all CPT-4 codes), including 23-h observations, in addition to procedures in free-standing ambulatory surgery centers.

Each database includes patient-level demographic and clinical data, such as age, sex, county of residence, race/ethnicity, International Classification of Disease-9 (ICD-9) or Common Procedure Terminology (CPT) procedure codes, ICD-9 diagnosis codes, and the patient’s expected source of payment. Before 2011, race data had substantial missingness. However, beginning in calendar year 2010, North Carolina law required all hospitals and ambulatory surgery centers to collect self-reported race and Hispanic ethnicity on all procedures and report these data to the database processors [14]. As a result, from fiscal year 2011 onward, race data were 98.9% complete in these administrative databases.

These data are available from the UNC Cecil B. Sheps Center for Health Services Research, but restrictions limit access to these data, which were used under a data use agreement for the current study and therefore are not publicly available. The data use agreement prohibits any “attempt to identify any specific individual (including, but not limited to patients, physicians, and other healthcare providers) who has been described or who may have been the source of the Data.” The agreement also prohibits “reporting any data with cell sizes of less than 10 individuals.” Data are available from the Sheps Center by academic and public health researchers upon application. Because the study data were de-identified and participant identification was prohibited, consent was not possible. This project was approved by the institutional review board of the University of North Carolina at Chapel Hill, which approved the study including a waiver of informed consent in compliance with all relevant UNC policies and state and federal laws.

### Exposure variables/identifying hysterectomy

For each surgery, the Discharge and Ambulatory databases list medical codes describing every procedure involved in the surgery (ICD procedure codes in Discharge database and CPT procedure codes in Ambulatory) and the reason for the surgery (ICD diagnostic codes). Hysterectomy and oophorectomy were classified using ICD-9 procedure codes in the discharge databases and CPT procedure codes in the ambulatory databases (Appendix A: Code List). The ICD-9 classification was consistent with standardized coding by the Health Care Utilization Project Clinical Coding System [15].

#### Exclusions

Our analytic sample was restricted to women aged 20 years or older with non-missing race data who resided in North Carolina at the time of surgery. We excluded any surgery with ICD-9 diagnosis codes associated with a gynecologic, breast, or gastrointestinal/rectal malignancy (Appendix A) or indicated as emergent, or urgent. As result of these restrictions, from our original dataset of 51,334 hysterectomies, 9435 surgeries were excluded from our final dataset for the following reasons: patient resided outside of North Carolina (*n* = 3030), sex was coded as male (*n* = 8), surgery was indicated as emergency or the result of trauma (*n* = 5), a diagnostic code indicated malignancy (*n* = 5812), patient was under age 20 years (*n* = 23), or information on race was missing (*n* = 557).

We estimated age-standardized rates of hysterectomy using data from the U.S. Census**.** First, we utilized census data to get counts of the female population of North Carolina in 2011, 2012, and 2013. Using these data as denominators for each respective year of surgery data, we estimated crude year-specific rates of hysterectomy. Next, to control for confounding by age, we produced rates which were standardized to the age distribution of US women in the year 2000 [16]. For the age-standardization, we calculated age-specific hysterectomy rates using five-year age categories: 20–24, 25–29, 30–34, 35–39, 40–44, 45–49, 50–54, 55–59, 60–64, 65–69, 70–74, 75–79, 80–84, 85+. Race-stratified hysterectomy rates for non-Hispanic Black and White women used race/ethnic specific population denominators and were standardized to the same year 2000 age distribution as the non-stratified analyses described above.

To evaluate whether rates of hysterectomy changed over time, differed by Black-White race among non-Hispanics, or differed by age group (20–39 years old, 40–49 years, 50+ years old), we fit Poisson models using generalized estimating equations methods, with age- and race/ethnic-specific county-level population as an offset term. Finally, we included interaction terms to determine whether secular trends or age effects differed by race. These interaction terms allowed us to evaluate whether Black women experienced a unique increase or decrease in surgery rates in compared to their White counterparts.

We assumed an unstructured correlation structure, using the Huber-White robust sandwich estimator, to account for clustering of the repeated measures on the same counties over time [17–19]. SAS 9.3 (Cary, NC) was used for all analyses, including calculation of 95% confidence intervals.

## Results

From 2011 to 2013, the overall number of hysterectomy procedures performed in North Carolina for benign conditions decreased 9.6%, from 14,648 in 2011 to 13,241 in 2013. Median age at hysterectomy was 44.0 years. The majority of women treated with hysterectomy were White (64.6%) or Black (28.3%) (Table 1). Most hysterectomies (67.6%) occurred in outpatient settings (Table 1). About half of surgeries were concomitant with bilateral oophorectomy (43.9%).

**Table 1:** Descriptive characteristics of hysterectomies performed in North Carolina in 2011–2013, stratified by setting (inpatient/outpatient)

	Overall N (%)	Inpatient N (%)	Outpatient N (%)
Total	41,899 (100%)	13,582 (100%)	28,317 (100%)
Race/ethnicity^a^			
Hispanic	1239 (3.0%)	513 (3.8%)	726 (2.6%)
White	27,072 (64.6%)	7225 (53.2%)	19,847 (70.1%)
Black	11,869 (28.3%)	5145 (37.9%)	6724 (23.8%)
American Native	767 (1.8%)	373 (2.8%)	394 (1.4%)
Asian	337 (0.8%)	119 (0.9%)	218 (0.8%)
Other	615 (1.5%)	207 (1.5%)	408 (1.4%)
Age			
20–29	1605 (3.8%)	552 (4.1%)	1053 (3.7%)
30–39	10,216 (24.4%)	3016 (22.2%)	7200 (25.4%)
40–49	19,795 (47.2%)	6075 (44.7%)	13,720 (48.5%)
50–59	6356 (15.2%)	2222 (16.4%)	4134 (14.6%)
60–69	2666 (6.4%)	1065 (7.8%)	1601 (5.7%)
70+	1261 (3.0%)	652 (4.8%)	609 (2.2%)
From procedure codes			
Hysterectomy only	23,044 (55.0%)	7898 (58.2%)	15,146 (53.5%)
Hysterectomy with bilateral oophorectomy	18,406 (43,9%)	5684 (41.9%)	12,722 (44.9%)
Hysterectomy – vague on oophorectomy^b^	449 (1.1%)	-.-	449 (1.6%)

^a^All women with Hispanic ethnicity were grouped together, and other race categories represent all non-Hispanic women

^b^Indicates procedure code definition allows for removal or retention of ovaries

**Fig. 1:**
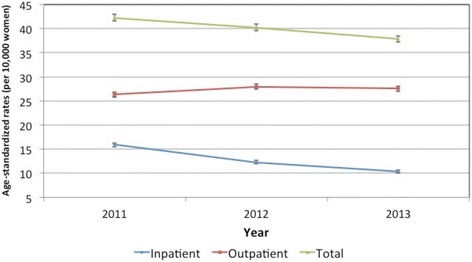
Age-standardized rates of hysterectomy performed in North Carolina in 2011–2013, stratified by setting (inpatient/outpatient)

Between 2011 and 2013, the age-standardized rate of hysterectomy decreased by 10.4%, from 42.3 per 10,000 women (95% CI: 41.6, 43.0) to 37.9 (95% CI: 37.2, 38.5) (See Table 2). Trends differed by setting (see Fig. 1). The age-standardized rate of hysterectomy in inpatient settings decreased by 35.2%, from 15.9 per 10,000 to 10.3 (see Table 2). In contrast, the age-standardized rate of hysterectomy in the outpatient setting increased from 26.3 per 10,000 in 2011 to 27.5 per 10,000 in 2013 (see Table 2).

**Table 2:** Hysterectomy rates (per 10,000 women) for North Carolina women aged 20 years and above, 2011–2013

Year	Crude rates	Standardized rates^a^		
		Rate	95% Normal Confidence Limits	
			Lower	Upper
Total hysterectomy				
2011	39.6	42.3	41.6	43.0
2012	37.4	40.3	39.6	41.0
2013	34.9	37.9	37.2	38.5
Inpatient hysterectomy				
2011	15.1	15.9	15.5	16.3
2012	11.6	12.3	11.9	12.7
2013	9.6	10.3	10.0	10.7
Outpatient hysterectomy				
2011	24.5	26.3	25.8	26.9
2012	25.8	27.9	27.4	28.5
2013	25.2	27.5	27.0	28.1

^a^Rates standardized using 2010 US Census age-specific population estimates

**Fig. 2:**
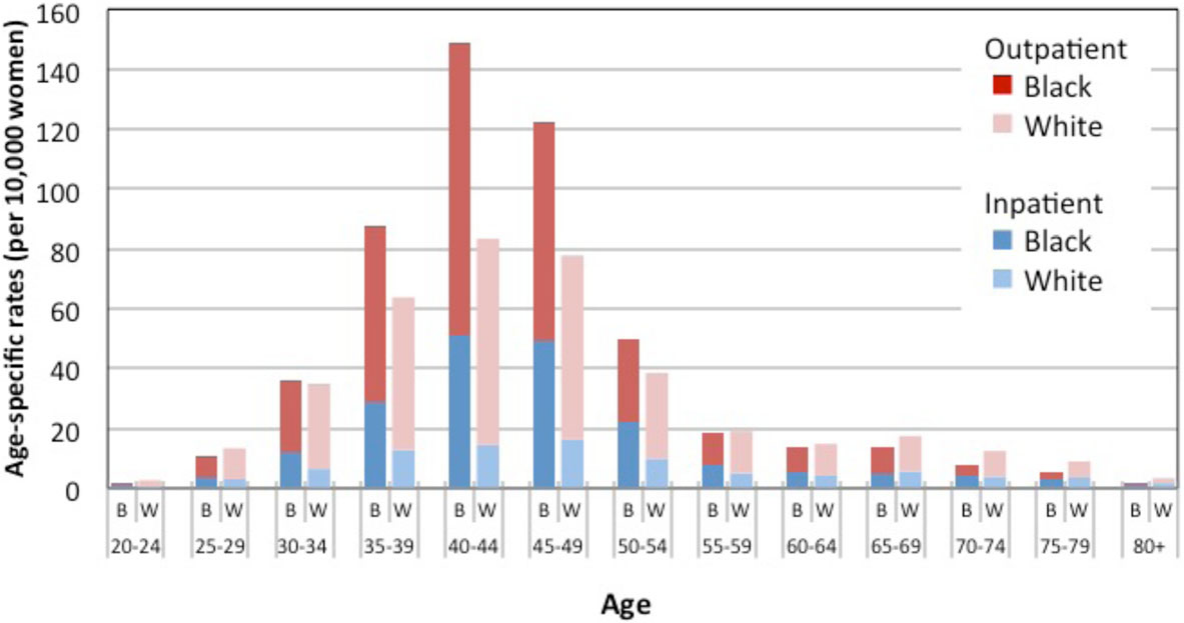
Age-specific rates of hysterectomy by race (non-Hispanic *Black* and *White*) in North Carolina stratified by setting (inpatient/outpatient), 2013

As seen in Table 3, age-standardized rates of overall hysterectomy were consistently greater among Black versus White women (*p* < 0.01 at all three years). In fact, from 2011 to 2013, the racial difference in overall hysterectomy rates grew. Among White women, overall rates of hysterectomy declined markedly: by 13.6%, from 41.9 per 10,000 to 36.0 per 10,000). The decline was much smaller among Black women, decreasing by 3.3%, from 51.5 per 10,000 to 49.9 per 10,000. Black women were also more likely than White women to be treated with hysterectomy at younger ages (*p* < 0.01) (see Fig. 2).

**Table 3:** Race-stratified hysterectomy rates (per 10,000 women), North Carolina, 2011–13

	Crude rates		Age-standardized rates^a^						Rate Ratio^b^ (ref = White)
Year	White	Black	White			Black			
			Rate	95% Normal Confidence Limits		Rate	95% Normal Confidence Limits		
				Lower	Upper		Lower	Upper	
Total hysterectomy									
2011	38.0	49.7	41.9	41.0	42.7	51.5	49.9	53.2	1.23*
2012	35.3	48.2	39.3	38.4	40.1	50.5	49.0	52.1	1.29*
2013	32.2	47.0	36.0	35.2	36.8	49.9	48.4	51.5	1.39*
Inpatient hysterectomy									
2011	12.2	25.0	13.1	12.6	13.6	25.8	24.6	26.9	1.96*
2012	8.8	20.4	9.4	9.0	9.8	21.1	20.1	22.2	2.24*
2013	7.2	17.5	7.7	7.3	8.0	18.5	17.5	19.4	2.40*
Outpatient hysterectomy									
2011	25.8	25.0	28.8	28.0	29.5	25.8	24.7	26.9	0.90*
2012	26.5	20.4	29.9	29.1	30.6	29.4	28.2	30.6	0.98
2013	25.0	17.5	28.3	27.6	29.0	31.5	30.2	32.7	1.11*

*p < 0.01

^a^Rates standardized using 2010 US Census age-specific population estimates

^b^Rate Ratios and *p*-values are from Poisson regression models

With regard to setting, between 2011 and 2013, rates of inpatient surgery decreased for both Black and White women but more dramatically for White women (Table 3). As a result, although the 2011 Black-White rate ratio for inpatient hysterectomy was already marked at RR = 1.96 (*p* < 0.01), two years later, this Black-White rate ratio had increased to RR = 2.4 (*p* < 0.01) (see Table 3).

In contrast, rates of surgery in outpatient settings increased by 22% among Black women (see Table 3). However, among White women, age-standardized outpatient rates remained stable (Table 3). As a result, between 2011 and 2013, Black women’s rate of outpatient hysterectomy surpassed that of White women (2011 RR = 0.90, *p* < 0.01; 2013 RR = 1.11, *p* < 0.01).

## Discussion

By 2013, 63% of Black women’s hysterectomy occurred in outpatient settings. The difference between this 2013 distribution (63%) and the distribution of outpatient surgery in 2011 (50%) may reflect unmet need of Black women who could have been good candidates for outpatient surgery in 2011. However, even with the spread of minimally invasive hysterectomy to all clinically appropriate women, some racial difference in hysterectomy setting may remain because of clinical differences between Black and White women. Without further research, however, it is unclear whether the saturation of outpatient surgery has been reached and what rate is appropriate in each group. However, the rapid increases in outpatient hysterectomy that we observed among Black women in a high-hysterectomy state at the same time that the rate has stabilized among White women indicate that some catch-up in the spread of minimally invasive hysterectomy is occurring among Black women, which is a positive sign indicating possible increasing equity in the adoption of hysterectomy in outpatient settings.

The rapid increases in outpatient surgery observed for Black women over a three-year period indicate that the spread of minimally invasive hysterectomy to all clinically appropriate women was still ongoing in the healthcare system for this group in the early 2010s. Among White women, stable rates of outpatient surgery indicate that this adoption of minimally invasive techniques for all eligible women may have already been achieved for White women by 2011. Taken as a whole, these results are consistent with the hypothesis that minimally invasive hysterectomy may have spread in use more slowly for Black women than their White counterparts after its introduction in the early 2000s.

The main driver of the choice of setting for hysterectomy is surgical approach. Minimally invasive techniques that translate well into an outpatient setting can result in less pain and shorter recovery time [2]. In addition, minimally invasive procedures lead to better body image satisfaction, sexual satisfaction, and overall quality of life [20]. Together, these benefits have led ACOG to recommend that benign hysterectomy be done in a minimally invasive fashion whenever possible [2]. Therefore, our results are encouraging, as we see a clear trend state-wide in increasing rates of outpatient surgery.

However, we found some indication that these improvements were not offered to all women equally. Black women in North Carolina experienced much higher rates of overall hysterectomy and inpatient hysterectomy than White women. In fact, the racial differences in these procedures actually grew larger over the two-year time period studied. In a cross-sectional analysis of data from 13 states, a recent HCUP statistical brief reported that Black and Hispanic women undergoing treatment for benign uterine fibroids more commonly had inpatient surgery whereas White women more commonly had ambulatory surgery [5]. These differences may be due to a combination of patient-level and system-level factors. Increased comorbidity in Black women may require longer hospitalizations, even in the setting of minimally invasive techniques. The increased burden of fibroids among Black women may result in Black women presenting with large uteri for which minimally invasive techniques are more challenging [4]. Future research that takes into account clinical factors including surgery type, size of uterus, BMI, and history of previous surgery will provide better understanding of racial differences in the adoption of less invasive surgical techniques.

Another mechanism that may contribute to racial differences in the spread of outpatient hysterectomy is that Blacks in the US may disproportionately undergo treatment at lower-resourced hospitals. Unfortunately, there is little research on racial hospital segregation in the treatment of benign gynecologic conditions. However, the literature on non-gynecologic procedures, including coronary bypass and lung cancer resection, indicates that Black patients are more likely to receive surgery at lower-resourced hospitals, less likely to receive care from board-certified physicians and high-volume surgeons, and less likely to have access to newer medical technology [21–23]. If these findings extend to hysterectomy, then Black women may be more likely to be treated in settings that are less likely to have expensive robotic platforms that may account for a large proportion of the rise in outpatient hysterectomy. They are also more likely than others to be on Medicaid or uninsured, which may be another pathway by which they are restricted from accessing robotic procedures that require more operating room time and more costly equipment. In addition, Black women may be less likely to have subspecialty-trained gynecologists with the greatest skill to perform more difficult minimally invasive procedures with the less expensive method of laparoscopy, even in the absence of robotics. Further research is needed to clarify these questions. Regardless of the mechanisms, if young minority women are more likely than others to be treated with hysterectomy for the same clinical conditions, then they are shouldering an unfair burden of infertility [24, 25]; surgical complications [4, 10, 26]; psychosocial harms [27, 28]; earlier age at menopause [29, 30]; and, if both ovaries are removed, immediate, intense menopausal symptoms and increased rates of mortality [31–34].

We also examined data on women of other race/ethnicities. The sample sizes for these groups were not large enough to support well-powered analyses stratified by year (see Table 1). However, we did observe differences in the proportion of outpatient surgeries for all non-White groups (see Table 1). Among White women, 73.3% of hysterectomy occurred in outpatient settings. For women classified as “other,” Asian, or Hispanic, these proportions were 66.3, 64.7 and 58.6%, respectively. Among Black women, the proportion was a little lower than among Hispanic women: only 56.7% of hysterectomy occurred in outpatient settings. However, the racial difference in setting was most pronounced among American Indian women, where only 51.4% of hysterectomy occurred in outpatient settings.

Our results confirm and update previous reports of higher overall hysterectomy rates among Black women, particularly at younger ages [4, 5]. Hysterectomy is the 2nd most commonly performed surgery among non-elderly U.S. women [1]. Our analysis confirm that age-adjusted hysterectomy rates were 39% greater among non-Hispanic Black versus White women in North Carolina (*p* < 0.01), with differences concentrated in women’s 30s and 40s, years of reproductive potential. Previous reports have expressed concerns about overuse of hysterectomy [35], especially given the high rates among U.S. Black women [7]. There is data also indicating higher rates among low-income women [11]. Analyses of self-reported data from the 1990s and early 2000s showed that Black women were treated with hysterectomy more often than White women [8]. In more recent data from inpatient surgery databases, race data were incomplete (typically missing on 30% of surgeries) [36]. Therefore, it had been unclear whether overall hysterectomy rates were changing differentially by race.

There are several possible explanations for the continued racial gap in overall hysterectomy rates. For one, hysterectomy rates vary by geography and might be 2.5 times as common in the U.S. South [8], where most (55%) Black women live [37]. This geographic patterning would result in higher rates for Black women nationwide even if rates did not vary by race within any given region or healthcare system. Second, racial differences in clinical need could explain the hysterectomy differences. For instance, Black women have higher rates of benign conditions (e.g., fibroids) that are indications for hysterectomy [4, 38].. However, the limited research on hysterectomy disparities finds that racial differences in diagnoses do not fully explain the differences in treatment with hysterectomy [7, 8]. In fact, there is evidence that hysterectomy is overused among poor and Black premenopausal women [7, 8, 10, 11, 39, 40].

Uterine-sparing treatments, such as oral conceptive pills, levonorgesterol-releasing intrauterine devices (IUDs), and myomectomy, provide alternatives to hysterectomy and are increasing in popularity but vary a great deal in cost and access [41]. Changing trends in treatment and the high level of variation in treatment cost and access for what are considered “discretionary treatments” tends to give rise to quality of care gaps that result in racial/ethnic and SES disparities [3]. Unequal treatment can become pronounced in several ways. When a procedure becomes disfavored or attractive alternatives are introduced [42], the older procedure’s use may be quickly “de-implemented” among the socially advantaged as newer alternatives spread more quickly to better insured patients. As a result, the older treatment can become increasingly concentrated among the less advantaged [3]. Alternatively, Black women, who are perceived to have more aggressive or unmanageable symptoms, may be steered towards more definitive but invasive treatments like hysterectomy. Future research should investigate racial differences in the use of non-surgical alternative treatment options that are less invasive and fertility-sparing [41, 43], while accounting for racial differences in indication and severity of underlying conditions.

Our work has several limitations that should be addressed by future research. First, we did not examine the extent to which racial differences in indication contributed to differential rates of hysterectomy in inpatient and outpatient settings. With claims-based administrative data, it is impossible to control for details of clinical indication such as fibroid size and patient symptoms. Therefore, we employed a modified difference-in-difference approach: given the short time frame examined, we believe that clinical indication would not have changed differentially by race and in dramatic enough a fashion to account for the trends we observed. In addition, we did not classify inpatient and outpatient surgeries by specific procedure type, which may be of interest in future work. Second, the results only generalize to North Carolina. To understand national trends, it will be necessary to examine surgery setting, trends, and racial differences in other states, especially those with historically lower hysterectomy rates and different racial/ethnic distributions. We also could not capture surgeries that North Carolina women experienced in other states, such as the neighboring states of South Carolina or Virginia. We also had limited statistical power to examine hysterectomy rates among Latinas, Asian, Native Americans, and other racial/ethnic groups. Finally, our analysis likely underestimated hysterectomy rates because the census denominators do not exclude women with previous hysterectomy. Underestimation will be more pronounced among groups with high hysterectomy rates of previous hysterectomy, such as older, Black women.

This research has important strengths. This was the first population-based study to examine racial differences in the shift of hysterectomy to outpatient settings. This analysis utilized a large, population-based dataset that included the universe of all surgeries performed in a single state. By restricting to one state, reduced confounding by geographic variation in clinical practice: we found that, even in a single high-hysterectomy state, Black women experience higher rates than other women. Additionally, we analyzed objective data from claims-based records, which have higher accuracy than self-reported data [44]. Further, we stratified by both age and race to compare racial differences in timing by age as well as setting. Finally, due to the large dataset with three years of coverage and linkage with census data, we were able to document population-based changes in surgery rates over time.

## Conclusions

This is the first study to provide evidence that minimally invasive surgical techniques may have spread more slowly to Black women, the racial group who experience the U.S.’s highest rates of hysterectomy. The persistently high rates of hysterectomy among young Black women and slower rate of adoption of outpatient hysterectomy highlight a potential racial disparity in women’s healthcare. Equitable use of less invasive surgical techniques has implications for equity in post-surgical care and patient-centered outcomes and satisfaction. Our findings point to the need for surveillance and analysis of gynecologic surgeries to monitor health system quality and racial equity.
